# A qualitative study of motivations for meditation in anthroposophic practitioners

**DOI:** 10.1371/journal.pone.0203184

**Published:** 2018-09-13

**Authors:** Terje Sparby, Ulrich Ott

**Affiliations:** Bender Institute of Neuroimaging, University of Giessen, Giessen, Hessen, Germany; Nord University, NORWAY

## Abstract

Research on meditation is advancing, but few studies about the motivations of meditators exist. Additionally, many forms and traditions of meditation have yet to be investigated. This study addresses both of these issues by presenting an overview of different forms of motivations found in contemporary Anthroposophic meditation practice. 30 Anthroposophic meditators were interviewed about their meditation experiences. The interviews were examined using thematic analysis. 14 data-driven themes were extracted and organized within a framework consisting of three superordinate theory-driven forms of motivation: External, internal and service. A developmental trajectory running from external and internal to service motivations is indicated. This approach improves upon a scheme developed by Shapiro by including additional types of motivations and being able to differentiate between forms of motivations that are fundamentally different: Self-related (heteronomous and autonomous) motivations and other-related motivations.

## 1. Introduction

Research on meditation, in particular mindfulness meditation, is advancing steadily. However, many other forms of meditation, such as Anthroposophic meditation, have yet to be investigated. Anthroposophic meditation is not well-known and has not previously been studied using the methods of qualitative content analysis. A full account of Anthroposophic meditation cannot be given here, as the range of practices is very extensive and so are their theoretical underpinnings. More in-depth overviews have been published elsewhere [1,2] and these will only be summarized here. Anthroposophic meditation arose in the early 20^th^ century. Its main features of are outlined in the basic writings of Rudolf Steiner [3–8] and expanded upon in different writings and lectures contained in more than 300 volumes. Characteristics that can serve as a way of differentiating Anthroposophic meditation practice are an emphasis on cognitive development (primarily thinking) and that it seeks to realize a higher self that is not ego-centered but still individual [9]. Furthermore, Anthroposophic meditation is often connected to other areas of human activity, such as investigating the natural world [10,11], and it is conceived of as providing support for pedagogy [12], medicine [13], agriculture [14], and religious practice [15]. For further characterization and historical contextualization of Anthroposophic meditation, see the commentary by Clement [16] and Sparby [1]. A typical Anthroposophic meditation consists of consciously activating different capacities of the mind, such as feelings, visualizations, and thoughts, in association with words or sentences. This is sometimes referred to as mantra meditation and is the most common form of meditation undertaken by the current sample[2].

Furthermore, certain aspects of meditation that may have an impact on the effectiveness and the actual outcome of the meditation practice, such as motivation, have received little attention. By investigating motivations for Anthroposophic practice, this article addresses both of these issues. Anthroposophy is increasingly becoming a topic of academic research [17–24]. Though there exists a handful of studies that address Anthroposophic meditation [10,25–30], the motivations that people have for meditating anthroposophically have yet to be examined. To our knowledge, this study is the first to address this issue. In combining a data-driven and theory-driven approach, it seeks to develop an initial overview of forms of motivation for Anthroposophic meditation practice today, to discover patterns of motivational change, and to compare Anthroposophic practice with other traditions and forms of meditation in relation to motivation.

What motivates meditation practice in general? Not much is known about this; there have only been few studies up until now [31–34]. Much more is known about the beneficial effects of meditation, in particular mindfulness meditation [35]. It should be obvious that all beneficial effects, or the idea of such effects, are potential motivators, though what these effects are varies greatly. However, some motivations may be more likely to provide the intended results, and, conversely, some motivations may be detrimental to obtaining particular effects. In addition, certain motivations may be supportive of establishing regular practice, which can be seen as a precondition the successful application of a meditative technique. Traditionally oriented and contemporary meditation manuals also put an emphasis on having a clear motivation and of regularly reminding oneself of this [36,37]. The level of adherence to practice varies considerably [38], and is related to adverse effects [39,40], personality, and other factors [31]. One meditation teacher is reported having said that he considered it a success if 5% of course participants were still meditating one year after taking an introductory course [41], and a study done Shapiro has found that there is indeed congruence between sought after and actual results, though perceived benefits can be both higher and lower than expected [31].

The same study [31] differentiates between three forms of motivations: Self-regulation, self-exploration, and self-liberation. While *self-regulation* includes stress and pain management, *self-exploration* includes personal insight and self-understanding, and *self-liberation* includes not only liberation from the ego, but also “developing a sense of harmony with the universe; the ability to increase one’s compassion, sensitivity and service to others” [31]. Together these are referred to as the SR-SE-SL continuum. This study also indicated that there is a shift from self-regulation to self-liberation on this continuum depending on the duration of practice. In Germany, a questionnaire has been developed by Schmidt and Netzt that based on a similar continuum: Well-being, self-regulation, self-exploration, self-transformation [42,43]. In applying this questionnaire it was found “that people with longer meditation experience have stronger spiritual motivation compared to more motivation for relaxation and wellbeing, which is more prevalent in short term meditators” [34].

A recent study of motivations for mindfulness meditation came to the conclusion that the only significant difference in motivation between meditators with less than 12 months of practice and those with more, was that the first group tended to be more motivated by reducing physical pain than the latter [33]. The results from the qualitative part of this study showed that spiritual reasons were slightly more likely to motivate commencing with practice (6.32%) than to motivate continuing with practice (4.23%), which can be seen to contradict Shapiro’s findings. However, this change in motivation is not significant, and may have to do with the more specific question of what motivates beginning with meditation and continuing with it. A further study of a group using mindfulness-based stress reduction to counter stress-related issues failed to reveal clear connections between different forms of intentions and outcomes [32]. However, there is evidence that certain forms of meditation, such as compassion training or metta meditation (also referred to as loving-kindness meditation, which consists of cultivating attitudes such as friendliness, gratitude, and benevolence towards others) themselves change people’s behavior and motivation towards being more pro-social [44]. Hence the answer to why there is a change in people’s motivation for meditation in the direction of becoming more spiritual (which includes compassion for Shapiro) might be that the meditation technique itself changes the practitioner. Shapiro’s study is the only one that has looked into differences between beginners and long-term practitioners, which is, of course, a prerequisite for uncovering overarching developmental patterns. Clearly, there is a need for further study within this area, not only to discover developmental patters and connections between motivation and effects, but also in order to be able to compare motivations between different traditions and forms of meditation. Further variables to investigate would be other forms of motivational shifts over time in relation to intensity and duration of practice, the influence of the worldview and conceptual framework of the practitioners, and also the sociocultural context in which the practice is undertaken (in a hospital, Eastern or Western monastery, solitary or group practice, etc.). However, since motivations are related to practice and different forms of practice may change the practitioners in a variety of different and unique ways, it may not be possible to generalize motivations across traditions and types of meditation.

## 2. Participants and methods

The design of this study was inspired by the “Varieties of Contemplative Experience” project [45,46]. Inclusion criteria for participants were: being at least 18 years of age, having Anthroposophic meditation as a main element of their meditation practice, having a regular meditation practice of at least one year, and having experienced at least one significant or unexpected effect of meditation, whether positive or negative. To ensure that the subjects could give accounts of a wide range of topics and phenomena, subjects with extensive experience in Anthroposophic meditation were purposefully recruited by approaching Anthroposophic meditators, face-to-face or by email, who were either friends, acquaintances, or colleagues of the authors. Further subjects were recruited with the aid of snowball sampling [47]. Thirty meditators of European origin were recruited (see Table 1 for demographic characteristics).

**Table 1:** Characteristics of sample (N = 30).

Demographic characteristics	
Female (%)	9 (30%)
Male (%)	21 (70%)
Mean age (years)	51
Average duration of regular practice (years)	ca. 20
European nationalities (%)	30 (100%)

The study design was approved by the local IRB at the University of Giessen, Fachbereich 06, Psychologie und Sportwissenschaft" (application 2014–0014). Written consent was obtained from all participants.

With two exceptions all interviews were conducted in person, and had a semi-structured open-ended format. The interviews were conducted by the first author (a male postdoctoral researcher), who received training by colleagues and collaborating researchers, and were carried out either German, English or Norwegian. Potential subjects were informed about the nature of the study both informally and through a standardized document; this included the participants being informed about the aim of studying more meditative traditions, such as Anthroposophy, and investigating the whole range of meditation experiences and effects, such as potential adverse effects. All interviews were audio recorded and the average duration of the recordings was about 1 hour and 40 minutes. After the audio recording of the interviews had been transcribed, a thematic analysis of the content was undertaken [48,49] using software (MAXQDA) for qualitative data analysis. The software helped establish the hierarchical order of the categories (see Table 2) and also to get an overview over which, and how often, motivations were connected to each other, either as different aspects of a compound motivation or in the form of a developmental trajectory. All participants were offered the opportunity to review and edit the transcripts. Three participants chose to do so. All interviewees were asked what their initial motivation for meditation practice was, and the analysis below is mainly based on their responses to this question. Major changes in the participants’ meditation practice became a topic in many interviews. Descriptions of the reasons for these were also included in the present study. Some subjects had experiences with other forms of meditation before coming to Anthroposophy. Responses that were not related to Anthroposophic practice were included as well, since they might give an indication of what kinds of motivations are representative of people who become attracted to Anthroposophic meditation. Additional questions were asked about any positive, negative, surprising or unexpected effects that the practitioners had experienced, but the responses to these were only taken into account in the present analysis insofar as they were deemed relevant to the question of what motivated their practice.

**Table 2:** Forms, themes and characteristics of motivations for meditation.

Form	Theme	Characteristics	Sample quotes
External (22)* (Heteronomous, non-reflected, indistinct, source “outside” the subject)	*Duty (3)*	What one should do, socially imposed expectations	“It was a little bit like a sense of duty.”; “It says there that one should do it.”
	*Disposition (2)*	Religious/spiritual attitude, indistinct calling, instinctual, related to childhood	“I had a religious disposition.”
	*Suggestion from others (1)*	Recommendation, meditation seen by others as an answer to a longing	“Yes, what, you are looking for is actually meditation. That’s something to try out.”
	*Non-reflected interest or curiosity (5)*	Spontaneous, naive	“Of course, it was to a certain extent curiosity.”
	*Needs and longings (7)*	Indistinct, self-imposing, pull, unconscious	"A kind of thirst."; “Somehow I had this need.”
	*Self-regulation (4)*	Coping, dealing with difficult emotions, facing up to the challenges of life, remaining balanced, pain management	“In order to keep myself balanced”; “To relax in order to avoid pain.”; “Because I wanted to find something that regulates strong emotions.”
**Internal (27)** (autonomous, reflected, articulate, source “inside” the subject)	*Developing higher capacities (11)*	Knowledge, verification, scientific attitude, first hand experience	“Because … I don’t just want to believe something, you want to experience for yourself. That is the attitude of natural science.”
	*Spiritual experience (4)*	Intensified perception, emphatic capacity, contact with supersensible reality, deeper meaning, connecting to truth	“Because I had kept on looking for that which I had experienced as a teenager”; “For me it was always about the deeper sense, the higher meaning, and the connection to truth.”
	*Self-realization or initiation (6)*	Sense of identity, revelation of the higher self	“I had the sense that it [meditation] had to do with me;” “Yes, I wanted to be initiated.”
	*Self-improvement (4)*	Self-discipline, develop character and virtues, existential core, devotion, equanimity, purification	“Because the question of self-improvement interested me. That one can create structure in the soul, that one can bring thinking into continuity, that one somehow can bring about emotional balance. That interested me. That it is possible to bring structure into the soul, thinking into continuity, and emotions into balance somehow.”
	*Incarnation (2)*	Becoming more embodied, anchoring in the world, grounding, stability	”Because the subsidiary exercises really are the anchor in the world for me.”
**Service (5)** (meditating for something or someone other than oneself)	*Gaining knowledge for the sake of practice (1)*	Understanding the spiritual nature of the human being, application, Eurythmy	"In order to understand eurythmy, you really have to understand the human being, and that means you have to understand its configuration”.
	*Service to the world and humanity (2)*	Will to help, make a difference, ethical mind-set, right motivation	"service to the world”; " And as I noticed that I really had the wish to help humanity, the people in my surroundings,–that I really have a will to help, that I really had highly pure intentions–then, finally, after months, the inner determination that now I’ll begin was there."
	*Realizing Anthroposophy (2)*	Meditation as foundation, actualization of Anthroposophy, avoiding irrelevance, establishing a community	"I sensed that if there are no one that works on this path of development as well as they can, then Anthroposophy will become a historical affair.

* This number represents the total number of instances of this form of motivation that were identified in the transcript

The method of analysis was both data-driven and theory-driven, both inductive and deductive. The reason for combining methods was that “there are no previous studies dealing with the phenomenon [motivations for Anthroposophic meditation]”, [50] which justifies an inductive approach, but also to “test a previous theory in a different situation”, [50] which requires a deductive approach. The themes were initially developed inductively based on the interview transcripts by the first author and then checked for validity by the second author. The 14 themes that were extracted were organized according to an overarching scheme influenced by Shapiro’s idea of an SR-SE-SL continuum and other motivational theories from psychology [51] and philosophy [52,53]. The divergence from Shapiro’s scheme were in part due to explanations the participants made of their own motivational shifts and in part due to implicit changes in motivation apparent when taking the biographical development of the participants as it was evidenced by the interviews into consideration. Additionally, some themes (such as duty, suggestion from others) did not fit into with Shapiro’s continuum, which led to a development of a new overarching scheme of external, internal and service motivations. Hence the analysis consisted of a dynamic process of interaction between the participant’s self-interpretation and the author’s interpretation of the participants’ statements, on the one hand, and employment of theory, on the other [54]. Once the overarching scheme was established, the transcripts where reviewed again, and this led to some minor revisions of how the themes were anchored in the transcripts, but not to major new discoveries. Some participants who were been present during initial presentations of the findings have provided feedback to the analysis, but this lead to no changes to it. Note that the different categories should not be considered to have clear-cut boundaries and other ways of organizing them may be possible. In particular, the distinction between internal and external motivations may be counterintuitive, since some motivations that are identified as external here can be considered as psychologically internal (for instance “needs and longings”) while others belong to the social environment (such as “suggestion by others”). It can be discussed to what extent the social environment is really distinct from the psyche, but this is beyond the scope of this article. The three categories presented below have the advantage that they enable the tracking of a specific form of psychological development, namely the one that is based on becoming reflectively aware of why one is doing something. Hence it is suggested that the reader understands external motivations as heteronomous, non-reflected, and/or indistinct, while internal motivations are autonomous, reflected, and/or articulate, as explained below.

## 3. Results and interpretation of specific themes: Motivations for anthroposophic meditation

Why do Anthroposophic practitioners meditate? As already indicated, three overarching forms of motivation were identified. One is a set of *external* motivations, where the practitioner describes the reason for why they meditate as something that comes to them from “the outside” and cannot be clearly formulated as a specific motif. External motivations contain such themes as suggestion from others, but also includes acting out of a (pre-)disposition or a given need (i.e. dispositions and needs that do not arise out of second order considerations of what kind of person the subject wants to be). Such motivations are opposed to *internal* motivations, where the practitioner has a clear idea of what the aim of their meditative practice is and how it relates to their own identity.

Examples of themes of internal motivations are self-development and self-improvement. The distinction between external and internal motivations drawn here based on the qualitative analysis is similar to the theoretical idea of extrinsic and intrinsic motivation [51], but mainly follows Kant’s distinction between heteronomous and autonomous acts [52]. An internally motivated meditator is more autonomous than someone who is motivated externally. However, external motivations can become internal, and certain motivations that seem internal can be different forms of introjected motivations. For example, duties imposed by social norms can be adhered to in an internal way if the subject is capable of giving reasons for these norms. Whether someone really identifies with these reasons is another question, and such an identification is a necessary condition for a motivation to be fully internal. In the current analysis care was taken to focus on what the subject stated while not speculating about which forms of internal motivations might be “impure”, i.e. introjected. The third from of motivation, *service*, is a combination of internal and external motivation: The subject is internally motivated to act for the benefit of someone or something else, which could also be considered a realization of freedom that goes beyond the distinction of heteronomy and autonomy [53], being fundamentally about relatedness [55]. Hence motivations can be seen as a process that forms a circle: They start out as external unreflected motivations, then become internally reflected motivations, and finally they are reflectively related to the external world again (other human beings, nature, etc.), which closes the circle. Again, care has been taken not to speculate about whether motivations of service are actually external motivations in disguise or varying degrees of introjected duties or norms. While themes are not mutually exclusive, external and internal forms of generally motivations are, although combinations are also possible.

It can be noted that no participant numbers are given in the following. This is to provide an extra level of anonymity; the Anthroposophic community is relatively small (there are about 47.000 members of the Anthroposophical Society worldwide [56]; the Anthroposophical Society in Germany has less than 14.000 members [57]). Though it is unknown how many people practice Anthroposophic meditation, participants of this study are likely to know or at least know of each other because of the snowball sampling that was utilized for recruitment. We expect more publications to come from this study and if participants numbers are used, it could potentially be possible to identify participants by coordinating numbers and quotations.

### 3.1 External

External motivations concern such matters as needs and longings that are not clearly articulated as a specific aim. External motivations also include duties that the participants do not identify with strongly, natural dispositions, and interests that the participants have not consciously reflected about or consciously identified with. These motivations are experienced as coming from the outside; they impose themselves on the subject, though not necessarily in a very forceful way.

This is in need of some clarification. Themes such as “duty”, “disposition”, and “needs and longing” may arguably be said to be “inside” the subject. However, they are *not* internal in the sense of being autonomous, reflected, and articulated; rather they are examples of being heteronomous, non-reflected, and indistinct. Hence an external motivation include such motivations that are given to the subject without it reflecting on how the motivation relates to its personal identity and larger life project; such reflection is part of internalizing the motivation. Take the example of a participant stating that the motivation was “a kind of thirst”. The thirst is indistinct (“a kind of”) and it is given; it is not part of a framework of reasons and beliefs consciously expressed or expressible by the subject that makes sense of the action. Such a framework potentially puts the motivation at the center of all the meaningful acts performed by it, giving expression to who the subject is as a person. “A kind of thirst” may be or may become part of such an framework (internalization). However, based on the interpretation of the descriptions provided by the participants in each particular case, all cases of external motivation are seen as not being embedded within such a framework. To take a further example, “a religious disposition” is not–at least not in this context–something that one has consciously developed, but rather something that one is born with or which simply presents itself (as a result of cultural condition perhaps, but that, again, would make it external). A religious disposition can probably be developed through conscious effort, but then it would be regarded as an internal motivation.

#### 3.1.1 Duty

Another external motivation concerns external duty, a sense that one should meditate, where the normative force of that sense is not internal to the subject. An example is of someone who was part of a study group of Steiner’s text states that the motivation was simply because it said in the text that meditation is something one should do.

A further example is of someone who decides to enter the *Freie Hochschule für Geisteswissenschaft*, an institution founded by Steiner and centered around a series of relatively long mantras. The reason for beginning to meditate was “a little bit the feeling that if I join [the *Freie Hochschule für Geisteswissenschaft*], then I also have to meditate. It was a little bit like a sense of duty”. It can be noted that the way it is formulated, the sense of duty was not particularly strong. But still, it is a matter of a duty that seems to come from the outside; at least it is not further articulated as directly connected to any internal motives. It can, of course, be discussed whether duties are an expression of autonomy or heteronomy, and here I will limit myself to saying that they can be both, depending on whether the person experiences the duty to belong to or embody what they are in a deeper sense.

A final example is someone who has the impression that the reason why people meditate within the Anthroposophic community is not so much a realization of their autonomy but rather a kind of non-reflected behavior within a social group:

And generally it seemed like […] it was kind of something they just did, and they did it because that’s part of what being an Anthroposophist was. […] I mean it’s a harsh thing to say, probably, but […] I didn’t get the feeling that they were becoming freer individuals.

This could of course be a matter of the subject not having insight into what the real motives of the other meditators were, but from the examples above it indeed seems possible to be motivated by duty in a heteronomous manner. In any case, it is implied here that meditation is supposed to make you more autonomous, more free.

#### 3.1.2 Disposition

When asked about the reason for why they started meditating, the interviewees sometimes went back to their childhood and spoke of a kind of disposition towards spirituality and spiritual practice. One subject mentioned “a kind of religious disposition” that he had always had, without it being tied up to any specific religion. Rather, it was described as “a form of inner intimacy tied up to a spiritual world”. Another subject described beginning spiritual practice at an age of four or five, and identified a kind of spiritual calling behind that. It seems then to be the case that some believe they are born with a certain religious or spiritual disposition that naturally or intuitively leads over into a form of practice, though the reasons for that remain unclear even in retrospect.

#### 3.1.3 Suggestion from others

Pepping et al. found that some people state that they started to meditate because it was recommended to them by someone else [33]. One such case was found among the Anthroposophic group of practitioners investigated here. One subject reported longing to come to a space beyond thinking, or a place where real thinking happens, and as a result of this, someone had said to him: “Yes, what, you are looking for is actually meditation. That’s something to try out, a meditation. It’s a fine thing.” The subject continued: “Like that, very non-imposing, without demanding it or something like that, but rather based on an understanding for what the longing is.” The other person recognized that meditation might be what the subject is longing for, so the motivation is in this case also related to satisfying a longing. It can be noticed that the suggestion or recommendation is not something the other person requires, but still, since the source of the motivation here is another person’s suggestion, the motivation is external to the subject.

#### 3.1.4 Non-reflected interest or curiosity

In some instances, subjects state that the initial motivation was not very reflected but rather spontaneous, or simply a matter of curiosity. One subject, who grew up in an Anthroposophic environment, said that “somebody would mention a particular exercise and so I’d just try it out. So it was just, you know, I’d just kind of pick something up and just decide, yeah, I’d give it a go”. Another subject says that “actually, the motivation came later, but yes, it was of course a little bit of curiosity, what is this thing that is described in for example “How to Know Higher Worlds?” [the most central work on Anthroposophic meditation [4,8] ]–can I enter into that? It was simply an interest”.

One subject suggested that initially the reason for trying the exercises was not very reflected, but rather something that happened naively and spontaneously, possibly out of a tendency or need to try out, or out of an enthusiasm for Anthroposophy. He also noted that the reason why he did not establish a regular or systematic practice was that it was not clear to him what his own motivation for meditation was. We can take this to mean that in order to establish a practice, one needs to find an internal motivation. An external motivation might be enough to make a commitment that one adheres to, in particular if there is no regulation of action coming from an outside source. It might be added that the subject also said the first attempts at meditation were “overrun” by the study of Anthroposophy and Steiner’s works. Strictly speaking, studying Anthroposophy is traditionally often conceived of as being the first part of the meditative path [5], so in that sense the practice had already started, though it was focused around assimilating the content of Anthroposophy as it is given in literature rather than applying a meditative technique.

#### 3.1.5 Needs and longings

This theme can be considered to be internal but is treated here in part because of how it is described (being pulled towards something) and also because “internal” is here taken to mean something that involves consciously formulating a motif. When asked about why he initially began to meditate, one subject stated that it was probably due to an unconscious pull towards it. Others mention a need or “a kind of thirst […]” that is met in meditation.

#### 3.1.6 Self-regulation

This theme is one that figures prominently in current meditation research. Meditation is known to have a wide variety of positive health benefits, like preventing relapse of depression [58] and enhancing well-being [35], and these are usually understood as forms of self-regulation [33]. In such context the motivation is anchored in a condition that is external and meditation is to some extent used instrumentally in relation to the external condition (though it can also be the case that the ultimate reason is linked to internal issues, such as when depression arises because of low self-esteem, which has been shown by several studies [59]).

Among the Anthroposophic practitioners interviewed for this study, no cases were found of someone beginning to meditate because of health reasons. However, one subject described wanting to find something to regulate her emotions. She stated that she had a constitutional disposition towards “feeling very strongly”, and that Anthroposophic practices could help with that. Another subject explicitly stated that her access to Anthroposophic practice came through coping with life (“Lebensbewältigung”). Another one said that establishing a practice came through dealing with becoming a father, which was described as being very challenging due to all the responsibility and continuous presence that is required. He began meditating regularly, as he stated, “in order to keep myself balanced. It was like that really, due to the circumstances of life”.

A more extreme case is someone who was beaten heavily as a child by a family member as part of her upbringing. She came across an article in a magazine about how to stop pain with the help of meditation techniques. At first it had no effect. However, on one occasion, it started working: “I remember that I was really naughty, I don’t remember what happened, but, you know, [I was] shouting or something. [The family member] said “stop shouting!” […] I remember that [she’d thrown] me down on [something] and she beat me on the back, as usual actually. And I remember that I’m lying down, […] and then I suddenly managed to do that processes [of meditation]. And this pain stopped. […] She stopped beating me. […] She said “are you not crying?”–she thought that she [had] beat me to […] death or something. She stopped beating me. She stopped beating me afterwards because it was such a strong experience for her as well.” Though it may seem inexplicable how a child can learn meditation from a magazine and manage to stop pain, it is well-known that meditation can help people to cope with pain. There have been several studies of meditation (MBSR) and pain management, and the effects are significant [60]. There have also been cases of self-immolation by monks where no reactions to the outside world are displayed (the most well-known case is probably that of the Vietnamese monk Thich Quang Duc). Hence at least the momentary cessation of pain through meditation seems possible.

### 3.2 Internal

Four types of internal motivations were identified: *Developing higher capacities*, *spiritual experience*, *self-realization or initiation*, *self-improvement* and *incarnation*.

#### 3.2.1 Developing higher capacities

This theme is the one that is most commonly mentioned (it was stated by 11 subjects). In the Anthroposophic literature on meditation and spiritual practice a range of new capacities of higher perception are described. Steiner’s books also contain numerous descriptions of higher worlds and beings. This can create a discrepancy between what meditators learn about from external sources and what they are able to experience for themselves. As one subject replied when asked why he wanted to try the Anthroposophic meditation practices: “Because … I don’t just want to believe something, you want to experience for yourself. That is the attitude of natural science.” Another subject elaborates on what seems to be a similar view:

I think it was the sense that Steiner was sort of more or less right about what he was describing. And that given that […] I thought he was right, then it was important […] to actually test these things, and kind of become conscious of them myself, rather than just believe him. […] I think I felt I wanted to verify and confirm the sort of things he was claiming.

These motivations concern what could be called a tension between faith and knowledge, of accepting or rejecting something based on whether one has seen or investigated matters for oneself. There is a sense of autonomy connected with experiencing things for oneself and the idea that one should follow a certain scientific procedure before committing to a belief. It is worth mentioning that Steiner claimed that Anthroposophy was scientific, that it developed out of the same attitude as natural science [5], so the subjects can be understood here as aligning themselves with intentions inherent in Anthroposophy. It should then be noted that this is not necessarily an expression of wanting to adhere to ideals given from the outside–which would likely be a case of introjected motivation–it could just as much be the case that they were initially attracted to Anthroposophy exactly because it aligns itself with a scientific attitude.

On several occasions subjects reported dissatisfaction with the state of Anthroposophic community when it comes to the way it relates to the content given by Steiner, i.e., as something taken on faith. This tension between the promise, intention or hope of spiritual realization, on the one hand, and its failures and ordeals connected with that endeavor, on the other, is one of the most central topics of the interviews. Subjects gave different accounts of how they dealt with it, and the details will have to be left aside here. Many, however, implicitly or explicitly take on the task of trying to enliven the core of Anthroposophy within a community where there neither is much evidence that the practices work, nor much interest in treating that as a problematic issue that needs to be discussed. At times they meet rejection from that very same community, sometimes they struggle to gain recognition, or find ways to justify continued practice without seeing results. The motivation of developing the capacities of higher perception of knowledge remains the same.

Still, there are examples of people describing being successful, and a slight variation of this theme is a case where meditation is motivated by developing capacities that are recognized as already present: “I also wanted, naturally, to develop the capacities that I already had.” A widespread view within the Anthroposophic community is that people will increasingly develop the capacities of higher perception/knowledge without having to practice meditation, but meditation can also be seen to support such capacities that are developing naturally. In any case, these are all examples of motivations where there is a clear idea of why one is doing the practice accompanied by a high degree of autonomy, and often a notion of scientific confirmation of spiritual beliefs.

#### 3.2.2 Spiritual experience

Two subjects described that they wanted to reconnect to a spiritual experience or religious sentiment from earlier in their life. One of them gave an account of a “very strong spiritual experience” he had had as teenager. It had started with restraining and detaching from emotions of happiness connected to being out in the country side, which then led to an intensified perception of nature and other human beings, a form of perception that was tinged with an impression of starting to learn something new. Later in the interview the same subject called this an emphatic capacity (#Einfühlungsvermögen#): “I had the sense that I could empathize really intensely with things and phenomena and people, and that is why it is so rich.” The state lasted for three months, slowly dissipating. This could be viewed as a form of extrovertive mystical experience, where a form of oneness is experienced through the physical senses and objects [61].When responding to the question of why he began meditating, he states: “Because I always sought again this experience that I’d had [in my teens]”.

The second subject interpreted his original motivation to be that he wanted reconnect to a form of childhood religiosity or spirituality. It can be noted that the motivation was not to revert to a childhood state, but rather to attain the same sense of being in touch with a reality beyond ordinary human senses that he had had as a child. This is true also for the previous report. The point there was not to relive a spiritual experience but to access the same rich emphatic perception that was characteristic of it. There is a connection here between the motif of re-accessing a spiritual mode of experience and the idea of developing new capacities of perception. Insofar as we understand “higher capacity” to mean a rich emphatic perception, then these themes are inherently connected. However, they can also be distinguished. The first (developing higher capacities) is associated with a scientific attitude and the notion of confirmation while the second one (spiritual experience) is devoid of that, and is characterized by being more personal.

Finally, it can be said that the sense of connecting with a spiritual form of experience always has to do with something that happened earlier in the life of the practitioner. It can also be about a spiritual connection more generally. As one subject formulates it: “For me it was always about the deeper sense of things, the larger meaning, and the connection to the truths”, though the latter aspect (connection to the truths) points in the direction of developing one’s capacities to know the spiritual world.

#### 3.2.3 Self-realization or initiation

This theme consists of a spectrum running from having a vague sense that meditation has something to do with who you are to a clear intention of fully revealing what you truly are as a human being through meditation. As one subject stated: “I had the sense that it has to do with me”. Another subject, who had grown up in an Anthroposophic family, was concerned with whether meditation really was something that had to do with him or rather was primarily due to his background. When asked whether he could remember his motivation for meditating, he responded:

There are different layers to that, but one was that I somehow sensed very strongly that I was inwardly identified with Anthroposophy. Because of that I really noticed that it was not only due my family background or something, but rather that it belongs to me. And then I began [meditating] very regularly, every morning in particular.

It is possible to understand “an inner identity with Anthroposophy” to mean that the subject saw himself as deeply connected with Anthroposophy and that meditation has got to do with him rather some external influence. This is a clear example of someone who seeks to establish that doing the practice is an autonomous activity, a topic we will return to below.

One of the most straightforward answers to the question of motivation was: “I wanted to be initiated.” The topic of initiation is a complex one, both generally and within Anthroposophy, and cannot be given an in-depth treatment here. Often initiation rituals have to do with a transition from childhood into adulthood [62]. Central issues are transition and death, but also gaining exclusive knowledge [63]. As Eliade describes it, someone who has gone through an initiation is “endowed with a totally different being from that which he possessed before his initiation; he has become *another* [64]. Eliade also states that initiations involve a series of ordeals that are an encounter with the sacred [64], which is a “reactualization of the primordeal event” [64]. This is the event that the mythology surrounding the imitation process describes as creating the cosmos *in illo tempore–*in the beginning of time; a time that is highly significant in that it gives meaning to all other events. In the specific initiation rituals of tribes, the initiands are told how the tribe came to be, and in different ways identify with the spiritual beings that were responsible for it. Additionally, Eliade states that “initiation is reducible to a paradoxical, supernatural experience of death and resurrection or of second birth” [65].

All of these elements can be found in Anthroposophy, but they take on a special significance. Steiner describes the process of initiation in the book *Wie erlangt man Erkenntnisse der höheren Welten*? (How to Know Higher Worlds) [4,8], but in many other places as well. Initiation in the Anthroposophic sense indeed consists of becoming someone completely different [66], of approaching the gate of death [66] and consciously going through what happens when someone dies [4]. This leads to the birth of a *higher self* [4], the person that one truly is. As part of this process, the initiand goes through a series of ordeals or tests. The highpoint of initiation consists of a meeting with a supersensible being that provides insight into the spiritual origin of the individual [4,5]. In other words, initiation in Anthroposophy could possibly be interpreted as a re-actualization of the primordial event of the creation of the *individual* human being rather than the cosmos or the tribe, etc. This is an issue that would require further elaboration. The point here is simply that the motivation for meditation stated by the subject is highly internal; it has to do with the deepest nature of the self, and an intention of coming to know it fully.

#### 3.2.4 Self-improvement

Three subjects either implicitly or explicitly stated that they were motivated by improving themselves, in the sense of developing their character and spiritual virtues such as devotion and equanimity. One subject was interested in self education (“Selbsterziehung”) at a young age, correcting herself when she made mistakes or told lies. She made lists of virtues, without knowing, as she underlines, anything about the monastic tradition, where such lists are common. In the Christian tradition we find for instance the tree of virtues, which is complimented by the tree of vices [67]. The development of specific virtues and overcoming of vices also often understood to be directly connected to meditative development, such as in Buddhist doctrine [68,69] and also in Anthroposophy [5]. One could even argue that meditation inherently is about developing virtues such as equanimity. One of the first Anthroposophic practices that the subject quoted above was the so-called subsidiary exercises (“Nebenübungen”), which is an example of a set of practices and meditations that both aim at developing specific virtues as well as developing higher capacities. When asked about why the subsidiary exercises had interested her initially, the subject in question answered: “Because the question of self-improvement interested me. That one can create structure in the soul, that one can bring thinking into continuity, that one somehow can bring about emotional balance. That interested me.” One of the practices of the subsidiary exercises consists of developing the ability to create and sustain a series of inferences relating to an object of choice, while other practices aim at fostering equanimity, positivity and openness. Each of the practices and their corresponding abilities or virtues are said to correspond to specific parts of the heart chakra [4].

Another subject mentioned that he wanted to develop devotion and inner calm (equanimity) described by Steiner in the beginning of *Wie erlangt man Erkenntnisse der höheren Welten*? (*How to Know Higher Worlds*) [4,8]. This practice became an existential core of his life, an expression of that which he really wanted to do. A further subject described how he was touched by the practice of the rosy cross that Steiner presents in his *Geheimwissenschaft im Umriss* (*Outline of Occult Science*) [5,7]. The meditation consists of imagining a human being next to a rose and comparing them. The plant grows in a dispassionate way in accordance with what it is, but is not free. The human being is free, but struggles with desires and has to purify itself. The subject says that he always pictured himself next to the plant, and that he entered into the feeling of the redness of the human blood that has to become purified before it can unfold itself like the blossoming rose. Here we see a connection between the notion of self-improvement in the sense of purification, catharsis, and the unfolding of what one is as an individual human being. In other words, the aim of self-improvement can also be seen to have to do with realization of what one truly is, and ultimately initiation.

#### 3.2.5 Incarnation

That meditation can have an incarnating (embodying) effect might be seen as surprising. However, one subject stated that her motivation for meditating was related not to entering the spiritual world, but rather the opposite. She sought to become an embodied inhabitant of this world, i.e., to incarnate. She reports having an initial negative experience with meditation, connected to a fearful out of body experience. This made her stop meditating actively for many years. When she started meditating again, the motivation was to become more embodied, and she chose specific exercises based on that, in particular the subsidiary exercises. Similarly, another subject reported becoming dizzy after using some of Steiner’s mantras, and then switched to the subsidiary exercises. The subject reported that he had a natural disposition towards dizziness, which was increased through mantra meditation. The subsidiary exercises did not have that effect. A final subject reported on a related issue. She had had strong spiritual experiences as a child, and stated that one of her main motivations in life was to incarnate, which initially made her cautious about meditation because she understood meditation to have mainly an opposite effect.

### 3.3 Service

A third overarching form of motivation consists of using meditation to help someone or realize something other than the meditating subject. These are motivations where the subject is not the main beneficiary of the practice, i.e., they are motivations of service. That which is sought to be realized can indeed either be in harmony with or potentially directly connected to the aims of meditation itself. Hence motivations like these are not necessarily purely instrumental, and in fact no examples of such motivations were found.

#### 3.3.1 Gaining knowledge for the sake of practice

A theme that did not come up often was the one of using meditation for some practical task or using it to gain knowledge that can serve as a foundation for practice (similar to how a knowledge of human physiology is applied by clinicians). However, this can be seen as one of the main features of Anthroposophy; applying knowledge gained through meditation in the world. There are, for instance, meditations for teachers and doctors that can be applied as part of their work [12,70,71]. Though one could imagine cases where someone comes to meditation in order to become a better teacher, no such cases were found. One explanation of this could be that the question of motivation was asked in the context of why the subject initially began meditating. And indeed there are examples of subjects beginning to use meditation within a school setting, so it could reasonably be said that the application of meditation becomes at least part of the motivation for meditation after someone has established a practice. There are many other examples of such applications. Some used meditation as part of preparing lectures, others may use it as a way of understanding the foundation of eurythmy (an Anthroposophic movement art with a therapeutic offshoot). One subject indeed states that this was her motivation, which justifies including it as a theme in this study.

#### 3.3.2 Service to the world and humanity

One subject mentioned that her practice was connected to having met someone with whom she had spoken about “service to the world”; she was “looking for a community of practitioners who wanted to make a difference, spiritually”. Meeting such a group was indeed what made her establish a very regular practice. This is included here in particular because relatedness to something outside of oneself arguably is one of the basic human motivators [55]. Another subject also understood the beginning of his regular practice as having to do with a wish of helping humanity. It had taken many years to develop this perspective. Being motivated in the right way, for him, had to do with altruism, helping humanity, and the people close to him. Egotism, including the wish to be regarded as somehow special or better than others, was considered the wrong, or morally impure, motivation. In a sense this is also about the subject making sure that the motivation is internal and not external. The wish for superficial social recognition would be a form of external motivation, which the subject apparently views as a wrong form of motivation. The proper motivation, helping others, is what leads to the inner determination of beginning a serious practice. Hence internal motivation does not necessarily exclude being motivated by service. That the subject is indeed internally motivated as well can be gleaned from the fact that he went through a long process of reflection about his motivation, rather than following some unclear external drive.

#### 3.3.3 Realizing anthroposophy

The final theme has to do with the idea that meditation is a way of realizing Anthroposophy. One subject stated that “meditation in many ways is what practicing Anthroposophy is all about”, and that meditation is “the foundation that in a way everything rests upon”. How this is to be understood is possibly explained best by another subject who was similarly motivated: “I felt that if there is no one that works on this path of development as well as they can, then Anthroposophy will become a historical affair.” The subject stated that he was drawn to Anthroposophy in part because of the things it does to transform the human life world, for instance through biodynamical agriculture (organic agriculture based on Anthroposophic ideas) and the Camphill villages (an Anthroposophic initiative based on providing support for adults and children with developmental challenges, mental health issues, or other special needs). This was seen as a result of Steiner’s abilities for knowing higher worlds, and he believed that what Steiner was missing was a scientific community and peers to discuss matters with. Without that it will not be possible to realize Anthroposophy further, and it will be reduced to something that only will interest an historian.

## 4. General interpretation of the themes

Sometimes different themes overlap. For example, a need to relax can be connected to self-regulation. In other words, different themes can function as parts of more complex motivations. The overarching forms of motivation can also be related to each other, especially over time. What starts as an external need or curiosity can become a clearly formulated intention of developing higher capacities or realizing oneself as a human being.

Though one could say that each and every case is highly individual, it is possible to notice certain patterns of development. A typical pattern seems to be a change from external motivation to internal. Here is an example: One subject stated that meditation for him was “somehow a need” and that it was connected with a “sense of meaning”. He also stated that as a child he had had the same deep sense of meaning when he went to church, something he did without anyone telling him to do so, but rather “because of an inner longing”. Here there is a mixture of internal and external motivations; on one level he went freely, i.e., autonomously, but behind all of this there was a longing for something (which could be construed as heteronomy). Most likely the deep sense of meaning had to do with the sense of meaning that was experienced in the church. The sense of meaning was not elaborated any further in the interview, but it could possibly be interpreted along the lines of being something that has to do with the self of the subject, so that the longing was for self-realization or even initiation.

As has been mentioned earlier, there are also examples of connections between having a disposition for meditation and spirituality and having had spiritual experiences as a child. Having a need or longing for something spiritual also can develop into a motivation for developing the higher capacities described in Anthroposophic literature, and the need can be connected to becoming more self-realized. There is also a case, which has been touched upon earlier, where an initial motivation of coping with life transformed into developing higher capacities and was combined with a wish for practically applying meditative skills for researching eurythmy. For some, self-regulation is a matter of self-improvement, and self-improvement can also be seen to be related to self-realization. Finally, a tendency also seems to be that in order to establish a regular practice, an external motivation has to become either a motivation that is internal or a motivation of service. For instance, there is one example of someone initially being drawn to meditation due to a disposition, while the practice becomes regular when it shifted to a motivation of service. A spiritual disposition could indeed be understood as a disposition towards service. Certainly, if we understand spirituality to mean a sense of connection to the whole out of which separate existence arises, then service could be understood as a way of embodying or manifesting spirituality.

Again, there is one clear overarching trajectory of development that goes from external motivation, through internal, to a motivation of service. The subjects often start out motivated by something that in one way or another is given from the outside. It can be a certain disposition towards meditation, a need or longing, simply curiosity, or a sense of duty. Sometimes external life circumstances or a tendency of having difficult emotions leads to using meditation as a way of self-regulating. This can be a way of self-improvement or of realizing one’s deeper nature, which is also related to the sense that one needs meditation or that one has a need to reconnect with the spiritual world. Often meditation is motivated by trying to open up an access to a spiritual world, which one hears of or has had some encounter with earlier, or simply has had a longing for. As the capacities start developing and a connection is established, the motivation can shift towards being about either realizing Anthroposophy, practically applying one’s capacities, or service to the world and humanity in general. This is then a way of manifesting the connection to the spiritual world within this world, a way of overcoming the situation where the spiritual is transcendent and not immanent; a further typical characteristic of Anthroposophic meditation and spirituality in general. There are examples of less ideal patterns of development, such as when meditation becomes an empty duty and a burden, but such aspects will have to be treated on another occasion. Though external factors may be an important part of the initial motivation for meditation, the tendency is that the motivation is internalized. This is, furthermore, in accordance with Steiner’s view that meditation is the only act that really is free within human life [72,73]. Hence there is a tension here between how the act of meditation is sometimes initially approached and how it was originally conceived. Furthermore, participants of this study have indicated that meditating out of duty, or similar external reasons, is both a source of psychological conflict as well as not being sufficient for establishing a regular practice. It can therefore possibly be helpful for beginning meditators who want to avoid this to reflect on their reasons for meditating, finding a way of internalizing the motivation, if it starts out as being external.

## 5. Discussion

Again, to our knowledge this is the first study of Anthroposophic meditation. 14 themes were extracted based on the data and were organized within an overarching framework consisting of external, internal, and service motivations. Certain motivations seem to be specific to Anthroposophy, especially when compared to mindfulness meditation. However, all forms of motivation identified by Shapiro can also be found among Anthroposophic practitioners. Furthermore, there are indications that the motivation for Anthroposophic practice gradually becomes more spiritual, but in ways that cannot be fully accounted for by the SR-SE-SL trajectory identified by Shapiro. The main reason for this might be that Anthroposophic meditators rarely seem to view meditation primarily as a way of self-regulation, but rather approach meditation with a spiritual understanding from the very beginning. Still, motivational shifts do happen, and it will be suggested that they follow a trajectory that proceeds from external motivations to internal motions and motivations of service.

It is possible to locate at least some of the themes extracted here on the SR-SE-SL continuum. Self-regulation (SR) has already been identified as one theme. Self-regulation can also be seen to include self-improvement, in the sense of developing character and virtue. However, self-liberation is sometimes seen to include personal growth, and hence self-improvement could also be located there. What is referred to here as self-realization is probably the theme that comes closes to Shapiro’s category of self-exploration (SE). Most of the themes extracted from the interviews would belong to Shapiro’s category of self-liberation (which also include service motivations, harmony with the universe, etc.): Spiritual experience, develop capacities of higher knowledge, realize Anthroposophy, service to humanity, practical application. In contrast, reasons such as disposition, duty, suggestion from others, and curiosity do not fit neatly anywhere, though the theme of need and longing can probably be understood either as a variety either of SE-motivation or SL since it sometimes is a longing for self-knowledge or a longing to connect to a spiritual world. The theme of duty can be seen as a motivation of service, which belongs to SL, though in the form it has been treated here, it is a matter of a duty that has not been internalized. The forms of motivation discussed in Pepping et al. 2016 [33] mostly concern SR, which makes further comparisons with that study difficult.

It seems apparent then that the way that motivations have been categorized in the research up until now is somewhat confused. However, this can be understood as a reflection of motivations are sometimes overlapping, or that they cannot be separated clearly from each other, or that they exist on a continuum. Furthermore, motivations need not always be very clear to the meditators themselves, especially in the beginning, and it should not be ruled out that motivations can also be expressive of an unconscious drive. What starts as a motivation of SR may on a deeper level be a matter of seeking a better understanding of oneself (SE), and SR, in the sense of reducing suffering, can be seen as the “light” version of SL (ending suffering once and for all). Indeed, in meditation self-exploration that leads to insight and liberation is often present at least implicitly. For example, meditation can bring to light how one is unintentionally creating stress through aversion; as this insight grows it might turn out that there is a drive towards the final end of suffering (liberation) inherent in the subject. Whether unconscious motivations for SE and SL are actually present cannot be decided here, but it could be a part of explaining why motivations shift on the SR-SE-SL continuum.

In order to study motivation for meditation better it should be clear that a more fine-grained and unambiguous system of classification is needed. In order to track connections between motivations and effects, shifts in motivations, etc., it is necessary to know more clearly what forms of motivations we are referring to. The overarching scheme that I have developed here improves on Shapiro’s in that it suggest a distinct line of development proceeding from external (heteronomous) and internal (autonomous) motivations to a unification of the two in service. In contrast, Shapiro’s scheme cannot differentiate between motivations of liberating oneself and compassion; both belong to SL. In the scheme developed here, self-liberation is an internal motivation while compassion would be service.

In other words, the critique of Shapiro’s scheme is twofold: Certain motivations do not fit into the scheme (such as duty, suggestion form others, etc.) and it fails to distinguish between basic forms of human motivation (such as self-centered and other-centered motivation). Both of these problems are remedied by the overarching scheme of the present study. Future research will need to investigate whether the external-internal-service line of development can be empirically supported. Furthermore, additional traditions need to be studied in order to discover the full range of motivations and other possible forms of motivational shift. Indeed, the ultimate aims of meditation may vary between traditions. Hence, clarity in the understanding of motivations for meditation cannot be gained without investigating of the full range of effects of meditation, including different models of enlightenment and liberation, and other contextual factors.

An obvious limitation to this study is the gender imbalance and that the participants come from European countries. A further limitation is that questions about the changes of motivation were not posed systematically and that the focus was on the initial reason for practice. This might explain why there are more external and internal forms of motivation in comparison to service. Future studies should take this into account. If the theory of a shift from external motivations to service is correct, it should be possible to identify more cases exemplifying the latter form.

What has been suggested here is that when systematizing motivations, one of the most fundamental questions is: Who is the intended beneficiary of the practice? Oneself or someone else? In this study the internal and external motivations are primarily self-related, while the motivation of service has others as its focus. One question that deserves further attention is what impact the experience of the dissolution or merging of self and other that is characteristic of some meditations, (such as loving-kindness) has on motivation for meditation practice and life in general. Additionally, it has been suggested that a main difference between motivations is that of heteronomy and autonomy, of unclear impetus (drive/disposition/curiosity) and conscious motif. Finally, it has been suggested that there is a development from heteronomous and autonomous motivations to service, corresponding to shifts on the SR-SE-SL continuum that other studies have indicated. However, further study, including statistical analysis of a much larger sample, is needed to investigate whether such a shift is typical or incidental in Anthroposophic practitioners, and whether the external-internal-service scheme can be found in other communities as well.
